# Supplementary material for: Quality of nutrition services in primary health care facilities of Dhaka city: State of nutrition mainstreaming in urban Bangladesh
Source: PLoS One. 2022 Dec 14;17(12):e0278621. doi: 10.1371/journal.pone.0278621 (PMC9749975; doi:10.1371/journal.pone.0278621)
Supplement: S1 Table — (PDF) [file pone.0278621.s001.pdf]

S1 Table: List of health facilities

Sl no.	Hospital name	Managed by	Located area
1.	Institute of Child Health & Shishu Sashtha Foundation Hospital	NGO	Mirpur
2.	MCH-FP Centre (Radda Plan)	NGO	Mirpur
3.	MCH-FP Centre (Radda Plan)	NGO	Mirpur
4.	MCH-FP Centre (Radda Plan)	NGO	Mirpur
5.	Surjer Hashi Clinic	NGO	Mirpur
6.	Surjer Hashi Clinic	NGO	Mirpur
7.	Surjer Hashi Clinic	NGO	Mohammadpur
8.	Surjer Hashi Clinic	NGO	Sabujbag
9.	Surjer Hashi Clinic	NGO	Razarbazar
10.	Surjer Hashi Clinic	NGO	Mirpur
11.	Surjer Hashi Clinic	NGO	Mirpur
12.	Surjer Hashi Clinic	NGO	Wari
13.	Surjer Hashi Clinic	NGO	Mirpur
14.	Surjer Hashi Clinic	NGO	Mohammadpur
15.	Surjer Hashi Clinic	NGO	Mirpur
16.	Surjer Hashi Clinic	NGO	Jatrabari
17.	Surjer Hashi Clinic	NGO	Mirpur
18.	Surjer Hashi Clinic	NGO	Badda
19.	Surjer hasi clinic	NGO	Mirpur
20.	Ad-Din Medical College Hospital	Private For Profit	Moghbazar
21.	Al Arafat Hospital	Private for profit	Mitford
22.	Al-Sami Hospital (pvt.)	Private For Profit	Badda
23.	Anwar Khan Modern Hospital Ltd.	Private for profit	Dhanmondi
24.	Bangladesh Association for the Aged & of Geriatric Medicine (BAAIGM)	Private for profit	Agargaon
25.	Bangladesh Medical College and Hospital-Dhanmondi	Private For Profit	Dhanmondi
26.	Care Medical College Hospital	Private for profit	Mohammadpur
27.	Dhaka Central International Medical College Hospital	Private For Profit	Mohammadpur
28.	Dhaka Pediatric-Neonatal & General Hospital Ltd.	Private For Profit	Mohammadpur
29.	Dhaka Renal & general Hospital	Private For Profit	Dhanmondi
30.	Dr. Azmal Hospital	Private For Profit	Mirpur
31.	Farazy Diagnostic & Hospital LTd	Private For Profit	Rampura
32.	Health Aid Diagnostic & Hospital	Private for profit	Sabujbag
33.	Holy Family Red Crescent Medical college Hospital	Private For Profit	Eskaton
34.	IBN Sina Medical college & hospital	Private For Profit	Kallaynpur
35.	Islami bank Central Hospital	Private for profit	Kakrail
36.	Islami Bank Hospital	Private For Profit	Mirpur

37.	Khidmah Hospital (Pvt.) Ltd.	Private For Profit	Khilgaon
38.	Life & care Hospital	Private For Profit	Hazaribagh
39.	Lubana General Hospital	Private For Profit	Uttara
40.	Monowara Orthopedic and General Hospital	Private for profit	Maniknagar
41.	Mother and Infant Health Care Hospital-Khilgaon	Private for profit	Khilgaon
42.	Nibedita Medical & Research	Private For Profit	Dhanmondi
43.	Pancare Hospital Ltd.	Private For Profit	Dhanmondi
44.	Redical Hospital	Private For Profit	Uttara
45.	Salauddin Specialized Hospital Ltd.	Private for profit	Tikatoli
46.	Seba Maternity Clinic	Private for profit	Cantonment
47.	Snehaloy	Private For Profit	Moghbazar
48.	United Hospital Ltd.	Private For Profit	Gulshan
49.	Uttara Central Hospital	Private For Profit	Uttara
50.	Selina General Hospital and diagnostic center	Private For Profit	Mirpur
51.	Shaheed Monsur Ali Medical College hospital	Private For Profit	Uttara
52.	Uttara Modern Medical College Hospital	Private for profit	Uttara
53.	Sasto Seba Cenro Badda	Private for profit	Uttor Badda
54.	Medilink Hospital LTD.	Private for profit	Badda
55.	Primary Health Care Centre-2	Private For Profit	Mirpur
56.	Al-Haj Johurul Islam Nagor Matrisodon Cenro-2	Private For Profit	Mirpur
57.	Zainul Haque Sikder Woman`s Medical College & Hospital (Pvt.) Ltd.	Private For Profit	Dhanmondi
58.	Bangladesh specialized Hospital Ltd	Private For Profit	Shaymoli
59.	Ashkona Clinic	Private For Profit	Uttara
60.	Fast Aid Hospital Ltd	Private For Profit	Kamrangirchor
61.	Medicare Diagnostic and Hospital	Private For Profit	Kamrangirchor
62.	Bangabandhu Sheikh Mujib Medical University (BSMMU)	Public Autonomous	Shahbag
63.	500 Bedded General Hospital, Mugda	Public Only	Sabujbag
64.	Bangladesh Railway Hospital	Public Only	Kamlapur
65.	BAVS Maternity	Public only	Mirpur
66.	Dhaka Medical College Hospital	Public Only	Chankharpul
67.	Kuwait Bangladesh Friendship Govt. Hospital	Public Only	Uttara
68.	Mohammadpur Fertility Services and Training Center (MFSTC)	Public Only	Mohammadpur
69.	Motijheel G .O. D Matrishodon Kendra	Public Only	Motijheel
70.	OGSB Hospital & Institute of Reproductive and Child Health (IRCH)	Public only	Mirpur
71.	Shaheed Shorowardy Medical College Hospital	Public Only	Agargaon
72.	Sir Salimullah Medical College (SSMC)	Public Only	Mitford
73.	Tofirunasha Praibaric Shastha Clinic	Public only	Nikunjo
74.	Sorkari Karmachari Hospital	Public Only	Tejgaon
75.	Kendreo Police Hospital	Public Only	Rajarbag

76.	Nagar Sastha Kendro-sabujbag	Public only	Sabujbag
77.	Nagar Sastho Cenro KMSS PA-2	Public only	Tejgaon
78.	Nagar Sastho Cenro KMSS PA-4	Public only	Motijheel
79.	Nagar Sastho-01(UTPS)	Public only	Darus Salam
