# Supplementary material for: Quality of nutrition services in primary health care facilities of Dhaka city: State of nutrition mainstreaming in urban Bangladesh
Source: PLoS One. 2022 Dec 14;17(12):e0278621. doi: 10.1371/journal.pone.0278621 (PMC9749975; doi:10.1371/journal.pone.0278621)
Supplement: S2 Table — (PDF) [file pone.0278621.s002.pdf]

S2 Table: Scoring of knowledge questions about nutrition counselling among health service providers

Sl no.	**Knowledge about topics of nutrition counselling should be discussed during ANC visit among ANC providers**	Response	Score
1	Animal source foods	Yes	1
		No	0
2	Vitamin A rich foods	Yes	1
		No	0
3	Fruits and vegetables	Yes	1
		No	0
4	Frequency of food consumption	Yes	1
		No	0
5	Iodized Salt	Yes	1
		No	0
6	Iron folic acid supplementation	Yes	1
		No	0
7	Calcium supplementation	Yes	1
		No	0
8	Early initiation of breastfeeding	Yes	1
		No	0
9	On exclusive breastfeeding for 6 months	Yes	1
		No	0
10	Dangers of introducing other liquids before 6 month of age	Yes	1
		No	0
	**Knowledge about topics of infant and young child feeding counseling should be discussed during pediatric visit among pediatric service providers**		
1	Initiation of breast feeding within an hour of birth	Yes	1
		No	0
2	Giving baby only breast milk and no other liquids or solids not even water for 1st 6 month of life	Yes	1
		No	0

3	Breastfeed more frequently if a mother thinks her baby is not getting enough breast milk	Yes	1
		No	0
4	Mother of a <6 month-old baby should not stop breastfeeding if the mother becomes ill	Yes	1
		No	0
5	A baby should be breastfed till 24 months	Yes	1
		No	0
6	A baby should first receive water or other liquid at the age of 6 month	Yes	1
		No	0
7	A baby should first start to receive semi-solid (e.g. rice, khichuri) food other than breast milk at the age of 6 month	Yes	1
		No	0
8	A baby should first start to receive animal source foods (e.g. beef, chicken, egg, fish) at the age of 6 month	Yes	1
		No	0
9	A mother should not stop breastfeeding if the child is ill	Yes	1
		No	0
10	A child >6 months with diarrhea requires zinc supplementation	Yes	1
		No	0
