# Supplementary material for: Quality of nutrition services in primary health care facilities of Dhaka city: State of nutrition mainstreaming in urban Bangladesh
Source: PLoS One. 2022 Dec 14;17(12):e0278621. doi: 10.1371/journal.pone.0278621 (PMC9749975; doi:10.1371/journal.pone.0278621)
Supplement: Attachment — Submitted filename: Response to reviewers.docx [file pone.0278621.s004.docx]

Rebuttal Letter

**Editor’s comments:**

1. Please ensure that your manuscript meets PLOS ONE's style requirements, including those for file naming. The PLOS ONE style templates can be found at
   <https://journals.plos.org/plosone/s/file?id=wjVg/PLOSOne_formatting_sample_main_body.pdf> and 
   <https://journals.plos.org/plosone/s/file?id=ba62/PLOSOne_formatting_sample_title_authors_affiliations.pdf>

**Author’s responses**: Thanks. We have addressed according to PLOS One style requirements.

2. You indicated that you had ethical approval for your study. In your Methods section, please ensure you have also stated whether you obtained consent from parents or guardians of the minors included in the study or whether the research ethics committee or IRB specifically waived the need for their consent.

**Author’s responses:** We addressed this in the method section of manuscript (line no 175).

3. We note that you have indicated that data from this study are available upon request. PLOS only allows data to be available upon request if there are legal or ethical restrictions on sharing data publicly. For more information on unacceptable data access restrictions, please see <http://journals.plos.org/plosone/s/data-availability#loc-unacceptable-data-access-restrictions>.

**Author’s responses:** Thanks. We shared the data set .

In your revised cover letter, please address the following prompts:

a) If there are ethical or legal restrictions on sharing a de-identified data set, please explain them in detail (e.g., data contain potentially sensitive information, data are owned by a third-party organization, etc.) and who has imposed them (e.g., an ethics committee). Please also provide contact information for a data access committee, ethics committee, or other institutional body to which data requests may be sent.

**Author’s responses:** We shared the data set.

b) If there are no restrictions, please upload the minimal anonymized data set necessary to replicate your study findings as either Supporting Information files or to a stable, public repository and provide us with the relevant URLs, DOIs, or accession numbers. For a list of acceptable repositories, please see <http://journals.plos.org/plosone/s/data-availability#loc-recommended-repositories>.

We will update your Data Availability statement on your behalf to reflect the information you provide.

**Author’s responses:** We shared the data set .

We note that you have included the phrase “data not shown” in your manuscript. Unfortunately, this does not meet our data sharing requirements. PLOS does not permit references to inaccessible data. We require that authors provide all relevant data within the paper, Supporting Information files, or in an acceptable, public repository. Please add a citation to support this phrase or upload the data that corresponds with these findings to a stable repository (such as Figshare or Dryad) and provide and URLs, DOIs, or accession numbers that may be used to access these data. Or, if the data are not a core part of the research being presented in your study, we ask that you remove the phrase that refers to these data.

**Author’s responses:** We have omitted “data not shown” phrase and we shared the data set

**Additional comments from Academic Editor:**

Comments to the author(s)
The "Quality of nutrition services in primary health care facilities of Dhaka City: state of nutrition mainstreaming in urban Bangladesh’ is an important and interesting manuscript that identified system-level gaps in nutrition service delivery. The study uses cutting-edge methods to analyze nutrition service quality in urban Bangladesh. Peer reviewers also found this paper interesting and suggested some modifications. I also have the following comments for better clarity of this paper:

a. Title:
   Authors may remove "primary" from the title as the paper considered both primary and secondary healthcare facilities (I see in table 1 that 20 secondary healthcare facilities are included in the analysis).

**Author’s responses:** We have removed the word “primary” from title.

b. Abstract:
   Please define ANC in the abstract. Please add findings with the sub-heading "Structural readiness", as they have for "process" and "outcome". Or take out all.

**Author’s responses:** We defined the word ANC in abstract (line no 31). We removed subheading in abstract. (line no 37 and 40).

c. Introduction:
   The introduction section does not adequately explain the existing urban nutrition services in Bangladesh. Readers may benefit from this description, please describe urban nutrition services in Bangladesh in line with the current Operations Plan for NNS.

**Author’s responses:** We have described urban nutrition services in Bangladesh in line with the current Operations Plan for NNS in introduction section (line no 65 to 70).

d. Methods:
   Please explain how the WHO/UNICEF NetCode protocol works for assessing health care facilities and how far the authors have used this protocol in this study.

**Author’s responses:** We addressed in the method section (please see line no 99-104).

e. Please add a functional definition of "quality nutrition services" as considered for this study.
   **Author’s responses:** Thanks. Please check line no 133-135.
f. Report how many service providers and mothers did not consent to being interviewed (non-response rate?), and how they were considered in the analysis, any statistical implications.
   **Author’s responses:** We reported this in the method section (line no 114-118).
g. Table 2 does not clarify the indicators adequately. In particular, what are the process and outcome indicators considered for this assessment?

**Author’s responses:** We added details of process and outcome indicators in the table 2 (line no 137).

h. Line 128: Please add a comma (,) after the guidelines. For supplements, please specify whether they are capsules or tablets, (e.g., Vitamin A capsule; Iron, Folic Acid (IFA) tablet, Calcium tablet)?
   **Author’s responses:** Addressed (line no 140).
i. Line 134-137: Please clarify what are the items of the 10 questions for calculating the knowledge score. How correct answers were assessed? Were they multiple choice questions or open-ended? I propose that the authors include a supplementary table to further clarify each of the knowledge items. I am also having trouble understanding how 10-questions’ knowledge scores ranged between 0-20, given that each correct question scored 1.
   **Author’s responses:** Thank you for your useful suggestion. We have added supplementary table for knowledge items (S2 table). We corrected the score range.
j. Line 153-54: Please specify the independent variables (e.g., type of health facility?) that are considered for multivariable modelling.
   **Author’s responses:** Addressed (line no 168-170).
k. Discussion:
   In the limitation, please acknowledge that the study did not fully comply with the WHO/UNICEF NetCode protocol and explain whether it affected the integrity of the study, if not how?
   **Author’s responses:** Thank you. We have addressed in the limitation section (line no 329-331).
l. Conclusion:
   Line 331: Use the acronym (MoHFW) as already defined earlier.
   **Author’s responses:** We addressed accordingly.
m. References:
   The references of the paper do not comply with the PLoS One recommended reference style. Please revise and format all references thoroughly.
   **Author’s responses:** We have checked and addressed accordingly.
n. Figures:
   Resolutions for both the figures (1A and 1B) need to be improved further. Please check
   PLoS One figure submission guidelines.
   **Author’s responses:** Thank you. We have addressed accordingly.
o. Overall, the paper needs careful proof editing for fixing some typos throughout the paper.
   **Author’s responses:** We have checked thoroughly and addressed accordingly.

[Note: HTML markup is below. Please do not edit.]

5. Review Comments to the Author

Please use the space provided to explain your answers to the questions above. You may also include additional comments for the author, including concerns about dual publication, research ethics, or publication ethics. (Please upload your review as an attachment if it exceeds 20,000 characters)

Reviewer #1: In this manuscript, authors have attempted to explore the quality of nutrition service provision at public primary health care facilities in urban Dhaka which is very timely research for Bangladesh. The findings of this study will guide the policy makers and implementer to plan interventions to improve the health services in the urban context. This is a very well written manuscript with sound statistical analysis.

A. Minor comments:
   Line 86: ‘From the list we identified health facilities that provided ANC and 87 postnatal care (PNC), delivery services, and pediatric care…’. It would be great asset for the readers if you add the list of health facilities as a supplementary file.

**Author’s responses:** We shared list of health facilities

B. Conclusion: I would suggest the author to provide specific, bold and focused 3-4 implications of the findings which could help the reader to have an idea about way forward and also to suggest some further research areas.

**Author’s responses:** Thank you for your useful suggestion. We addressed this (line no 346-348).

Reviewer #2: This research article is an effort to fill gap in the knowledge base of quality of nutrition services provided by primary and secondary healthcare providers in Dhaka city and reveals the state of nutrition services in urban setting in Bangladesh. I would recommend to accept the article for publishing, with subject to the following minor revisions:

1. In the title and abstract, you described that this research is about scrutinizing the primary healthcare services, however, you have taken samples from the secondary healthcare providers also. This should be addressed throughout the manuscript consistently.

**Author response:** Thank you. Though we used sample from secondary health facilities but we mainly focused on primary health care service delivery in those secondary health facilities.

2. In methodology part, you said about multivariate analysis, but did not say particularly which sort of multivariate analysis was performed. For example, I guess there were two different models (possibly linear regression with factor variables, not mentioned explicitly) for two different type of respondents, as they have different set of independent variables. These need to be elaborated more precisely.

**Author’s responses:** We addressed in method section (line no 169-171).

3. In describing the multivariate analyses, the authors mentioned about Table 8, which is non-existent.

**Author’s responses:** We addressed.

4. Regarding the tools, you said that minor adjustment in the context of Bangladesh has been made. However, the source tools were not described explicitly, let alone mentioning about the adjustment in specific.

**Author’s responses:** Spring tool was used as our source tool. Reference has been given in line no 128.

5. In the introduction of abstract (line 26) there is mention of only public facilities, whereas, in results section (line 42) there are mentions of public, NGO and private service providers.

**Author’s response:** We addressed accordingly.

6. In discussion section, while describing the structural readiness of the providers, you described the percentage of facilities or receivers (line 273-276) having the services or about receiving the services, respectively. I would suggest to mention the figures in reverse (mentioning the % of negative side), because only then the bigger gap with bigger number would be more visible to the reader or policy makers.

**Author’s responses:** Thank you for your feedback. We have addressed as per your feedback (line no 282-284).

7. In discussion (line 306), you mentioned about Sustainable Development Goal 2, but did not mentioned about the goal in few words. I think, for general readers, it should be mentioned in text. Further, the goal in general is about ending hunger, achieving food security and improving nutrition. So, you should mention specifically which sub-goal you intended to mention.

**Author’s responses:** We have addressed accordingly (line no 361).

8. One of the missed opportunity in the discussion section is that there is absence of illustrating the outcome in the light of multivariate models. Rather, bi-variate results were mainly explained. For example, the authors could mention about that the pregnant mothers are sensitive on their satisfaction level (significantly) if under age 19 or over age 25. Further, satisfactions are significantly reducing while the recipient are receiving them from the Government facilities (p<0.001). These results and their policy implication should be reflected in the discussion section.

**Author’s responses:** Thank you for your suggestion. We have addressed this in the discussion section (line no 314-317).

9. Finally, I would prefer the graphs with data value labels for the bars/columns.

**Author’s responses:** Data value labels has been added.

6. PLOS authors have the option to publish the peer review history of their article ([what does this mean?](https://journals.plos.org/plosone/s/editorial-and-peer-review-process#loc-peer-review-history)). If published, this will include your full peer review and any attached files.


If you choose “no”, your identity will remain anonymous but your review may still be made public.


**Do you want your identity to be public for this peer review?** For information about this choice, including consent withdrawal, please see our [Privacy Policy](https://www.plos.org/privacy-policy).

Reviewer #1: No

Reviewer #2: **Yes:** Kabir Ahmad

[NOTE: If reviewer comments were submitted as an attachment file, they will be attached to this email and accessible via the submission site. Please log into your account, locate the manuscript record, and check for the action link "View Attachments". If this link does not appear, there are no attachment files.]

While revising your submission, please upload your figure files to the Preflight Analysis and Conversion Engine (PACE) digital diagnostic tool, <https://pacev2.apexcovantage.com/>. PACE helps ensure that figures meet PLOS requirements. To use PACE, you must first register as a user. Registration is free. Then, login and navigate to the UPLOAD tab, where you will find detailed instructions on how to use the tool. If you encounter any issues or have any questions when using PACE, please email PLOS at [figures@plos.org](mailto:figures@plos.org). Please note that Supporting Information files do not need this step.
